# Malignant Mesothelioma of Tunica Vaginalis Testis: Macroscopic and Microscopic Features of a Very Rare Malignancy

**DOI:** 10.7759/cureus.1860

**Published:** 2017-11-19

**Authors:** Ersan Arda, Mehmet Gürkan Arıkan, Gizem Cetin, Uğur Kuyumcuoğlu, Ufuk Usta

**Affiliations:** 1 Urology, Trakya University Medical Faculty; 2 Anesthesiology, Trakya University Medical Faculty; 3 Pathology, Trakya University Medical Faculty

**Keywords:** malignant mesothelioma, tunica vaginalis testis

## Abstract

Malignant mesothelioma of the tunica vaginalis testis (MMTVT) is an extremely rare tumour, usually mimicking benign pathologies of the scrotum.

Our case is an 84-year-old male patient who appealed with a painless, left-sided scrotal swelling longer than 2 months. Although the level of tumour markers was normal, ultrasonographic examination results forced us to perform an inguinal scrotal exploration. Multiple small papillary tumours, both on tunica vaginalis and tunica albuginea, were detected intraoperatively. Due to these findings, radical orchiectomy was performed.

A pathological evaluation showed malignant mesothelioma (MM) of the tunica vaginalis testis. Exposure to asbestos is a well-known risk factor. Furthermore, a history of trauma, herniorrhaphy and chronic hydroceles is blamed as a possible risk factor. Scrotal ultrasonography is the mainstay of primary diagnosis and, therefore, it should not be overlooked when dealing with benign scrotal cysts or hydroceles, which are very common pathologies at these decades, too.

Radical inguinal orchiectomy is the primary treatment choice for localised MMTVT disease, whereas in signs of lymph node metastasis, inguinal lymph node dissection is required. Radical resection should be completed with chemotherapy and/or radiotherapy for an advanced or recurrent disease. This case, which is very rarely reported in the literature and detected during inguinal exploration, along with the pathological works that supported the diagnosis, was presented with this report.

## Introduction

Malignant mesothelioma of the tunica vaginalis testis (MMTVT) is an extremely rare tumour, usually mimicking benign pathologies of the scrotum [1]. Mesothelioma usually arises from the serosal cells of the pleura and peritoneum. Scrotal mesothelioma comprises less than 1% of the cases [2-3]. Most cases are reported between 55 and 75 years of age, while 10% of the cases are seen in patients younger than the age of 25 [3]. Due to its rare nature, its epidemiology and risk factors are still not well established. However, asbestosis, which is a well-known risk factor for mesothelioma, is only reported in 30%-40% of such cases [4]. Additionally, a history of trauma, herniorrhaphy, and chronic hydroceles are blamed as possible risk factors [3].

## Case presentation

**Figure 1:**
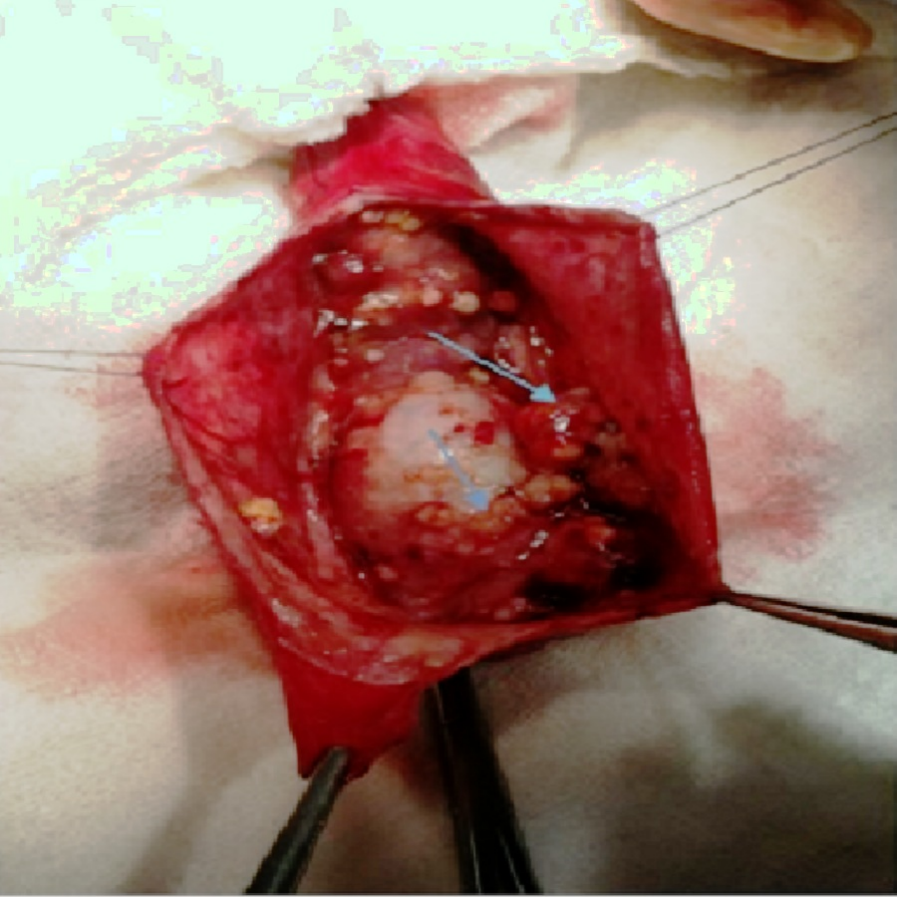
Intraoperative view of tumoural masses

We are presenting an 84-year-old male patient who appealed with a painless, left-sided scrotal swelling longer than 2 months. The right testis was normal and no inguinal palpable lymph node was detected. Also, there was no history of previous surgical intervention or trauma to this region reported by the patient. An ultrasonographic evaluation demonstrated irregular thickening and well vascularized, multiple papillary solid lesions of the tunica vaginalis. Testicular tumour markers, alpha-fetoprotein (AFP) and beta human choriongonadotropin (B HCG) levels were within normal ranges. A computed tomography (CT) of the pelvis, abdomen and thorax showed no sign of metastasis to solid organs to lymph nodes. Although a normal level of tumour markers was detected, ultrasonographic examination results caused an inguinal exploration. The skin was incised 2 cm above the inguinal ligament from the deep ring to the superficial ring and the inguinal canal was detected. After clamping the testicular cordal structures, tunica vaginalis was exposed from the adjacent structures. When it was incised vertically, multiple small papillary tumours, both on the tunica vaginalis and the tunica albuginea, were detected (Figure 1).

**Figure 2:**
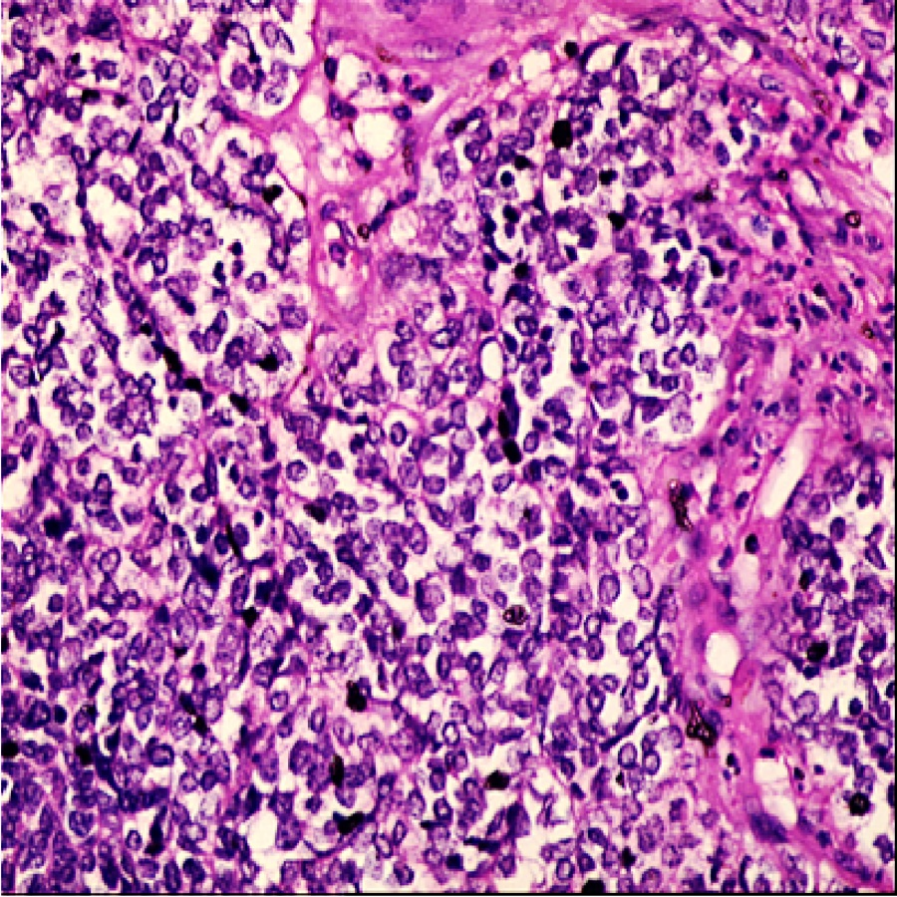
Pathological evaluation with haematoxylin-eosin

According to these intraoperative findings, a radical orchiectomy was performed. A pathological gross examination of the specimen showed multiple, papillary, tan/white-coloured nodulations on the parietal and visceral leaves of the tunica vaginalis, with an average size of 0.5-1 cm. Similar papillary masses on the outer surface of the tunica albuginea were also found. Macroscopically, the testis showed the normal structure of the parenchyma and no tumour invasion to the tunica albuginea was determined. A microscopic evaluation showed a solid and nesting pattern, which was composed of atypical epithelioid cells with clear and eosinophilic cytoplasms. The nuclei of the tumour cells were pleomorphic and hyperchromatic, with prominent, big nucleoli (Figure 2).

**Figure 3:**
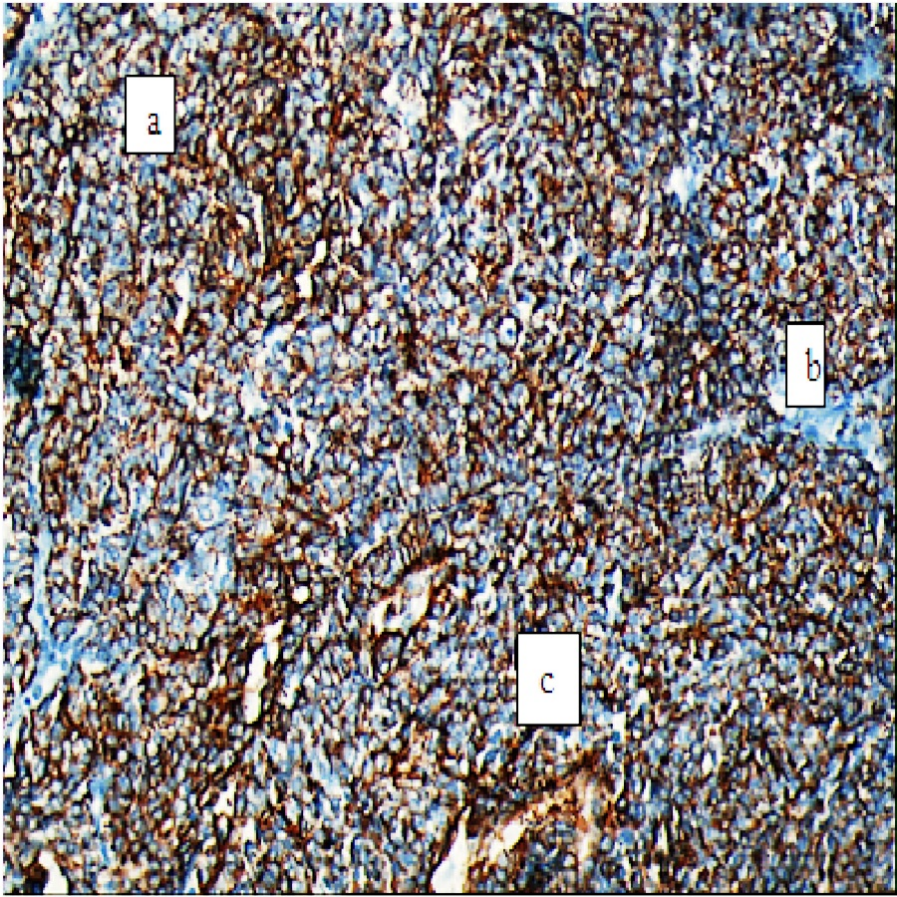
Pathological evaluation with immunohistochemical pattern

Immunohistochemically, tumour cells were extensively positive with calretinin and creatine, focally positive with epithelial membrane antigen (EMA) and showed a focal nuclear reaction with WT-1. But, it was negative with vimentin, cytokeratin (CK5), carcinoembryonic antigen (CEA) and B72.3 (Figure 3).

## Discussion

Less than 300 cases of MMTVT, with poor information of long-term follow-up and multimodal therapy, have been reported [3-7]. Asbestos exposure is a well-known risk factor. Furthermore, a history of trauma, herniorrhaphy and chronic hydroceles are blamed as possible risk factors [3]. Thirty-two point four percent (32.4 %) of patients with mesothelioma of the tunica vaginalis were detected with asbestos exposure. These outcomes showed consistency with the results, which indicated a positive job-related history in 41% of the 27 cases reviewed by Jones et al. [3]. Although, we didn’t identify any risk factor in our case, it is an aggressive tumour, often related to poor prognosis and high rates of distant metastasis and mortality [5]. The median survival was found 4-18 months for pleural mesotheliomas and 23 months for scrotal mesotheliomas [3]. This difference was thought to be because of early diagnosis in scrotal types, which is possibly easier, as it's more superficial than the other ones located intrathoracically. According to its very low prevalence, no guideline for treatment has been established. Therefore, early diagnosis and radical treatment seem crucial [6]. Unfortunately, the majority of cases are not diagnosed preoperatively because of nonspecific presentations [3]. In only two cases (2.7%), a correct preoperative diagnosis was obtained. Indications for surgery most frequently stated as a rapidly growing hydrocele (56.3%) or a suspected testicular tumour (32.8%) [3]. Diagnosis is mainly based on radiologic modalities (especially scrotal ultrasonography) since it is almost impossible to distinguish hydroceles during a physical examination [7]. Computed tomography should be performed to diagnose metastasis or any pathological lymph node [8]. Radical inguinal orchiectomy is the primary choice of treatment for localized MMTVT, while in signs of lymph node metastasis, inguinal lymph node dissection is required. For advanced or recurrent disease, radical resection with chemotherapy and/or radiotherapy is advised. Additionally, cisplatin and doxorubicin have been suggested for chemotherapy [8]. Patients should be advised to consult experienced multidisciplinary cancer centres for a second opinion on histology and the treatment plan. A regular follow-up plan is essential for the early diagnosis of recurrence, which is more frequent in the first two years after diagnosis [9]. Overall recurrence was found to be 52.5% and more than 60% of recurrences occurred within the first two years [3]. Our patient did not receive chemotherapy or radiotherapy because a radical inguinal orchiectomy was performed at an early stage, and the patient showed no sign of metastasis or recurrence.

## Conclusions

MMTVT should not be overlooked when dealing with benign scrotal cysts or hydroceles, which are very common pathologies in the same decades. Also, aggressive surgery and adjuvant therapy are necessary to achieve long-term survival.

## References

[1] Menut P, Hervé JM, Barbagelata M, Botto H (1996). Bilateral malignant mesothelioma of the tunica vaginalis testis. Apropos of a case [Article in French]. Prog Urol.

[2] Attanoos RL, Gibbs AR (2000). Primary malignant gonadal mesotheliomas and asbestos. Histopathology.

[3] Plas E, Riedle CR, Pfluger H (1998). Malignant mesothelioma of the tunica vaginalis testis: review of the literature and assessment of prognostic parameters. Cancer.

[4] Bisceglia M, Dor DB, Carosi I, Vairo M, Pasquinelli G (2010). Paratesticular mesothelioma. Report of a case with comprehensive review of literature. Adv Anat Pathol.

[5] Janssen-Heijen ML, Damhuis RA, Klinkhamer PJ, Schipper RM, Coebergh JW (1999). Increased but low incidence and poor survival of malignant mesothelioma in the southeastern part of the Netherlands since 1970: A population-based study. Eur J Cancer.

[6] Pelzer A, Akkad T, Herwig R, Rogatsch H, Pinggera GM, Bartsch G, Rehder P (2004). Synchronous bilateral malignant mesothelioma of the tunica vaginalis testis: early diagnosis. Urology.

[7] Mak CW, Cheng TC, Chuang SS, Wu RH, Chou CK, Chang JM (2004). Malignant mesothelioma of the tunica vaginalis testis. Br J Radiol.

[8] Gupta NP, Kumar R (2002). Malignant gonadal mesothelioma. Curr Treat Options Oncol.

[9] Mrinakova B, Kajo K, Ondrusova M, Simo J, Ondrus D (2016). Malignant mesothelioma of the tunica vaginalis testis. A clinicopathologic analysis of two cases with a review of the literature. Klin Onkol.

